# Medico-Chirurgical Transactions: Mr. Earle on Paraplegia, &c.

**Published:** 1828-04-01

**Authors:** 


### IX.
### MEDICO-CHIRURGICAL TRANSACTIONS.

### 1. *Mr.* Earle *on Paraplegia.*

In the last volume of the Medico-Chirurgical Transactions, Mr. Earle has occupied nearly 50 pages with the subject of paraplegia, respecting the pathology of which there has been a good deal of discussion, since Dr. Baillie's paper appeared, about 7 or 8 years ago, in the College Transactions. It is well known that Dr. B. believed paraplegia to be most commonly dependent on disease of the brain, when happening in adults, and when not evidently connected with outward violence to the spine. Dr. B. was not aware that Mr. Earle had published some cases, five years previously, in the Edinburgh Journal, shewing that paraplegia does sometimes depend on cerebral, and not on spinal disease. When we remember that sense and motion are lost the moment that an interruption takes place in the nervous communication between the brain and the part paralyzed, whether the interruption be seated in the medulla oblongata, the spinal marrow, or any portion of the nerves leading to the muscle or skin, we see no occasion for these prolonged discussions respecting the seat of the obstructing cause, nor any reason for pride in finding paralysis of the lower half of the body in people who have organic disease of the spine. It is strange that these controversialists should not dream, that the immediate cause of the paralysis may be a change in the nerves of the part paralyzed, without any disease in either brain or spinal marrow. Does the circulation never fail, unless there be disease of the heart or of the aorta? We see paralysis, for example, in both the feet, or in both the hands, while the muscular power and sensibility remain in all other parts of the body. How, we would ask, can this depend solely on disease of the brain or of the spinal marrow? Can the brain or the spinal marrow say to the nerves going to the lower extremities, you shall carry our motive commands to the muscles of the thighs; but whenever you pass the knee-joint, you shall no farther be the bearers of our despatches? Or, can they say to the other constituents of these nerves, we will acknowledge the receipt of all impressions made on the thighs, but if you bring impressions from the legs or feet, we will not receive them? The thing is preposterous. But, if we allow that those portions of nerves which are below the knees are incapable, from some morbid change in themselves, of transmitting the will from above, or sensation from below, then all is clear and intelligible. What, after all, is the spinal marrow, but a prolongation of the brain? What are the nerves, but prolongations of the spinal marrow? Ergo, what is the minutest and most invisible point in which a nerve terminates, but a portion of the brain? It is so with the vascular system. The minutest capillary is as much entitled to the appellation of a sanguiferous vessel as the great aorta. Disease in any branch of the immense tree of circulation will disturb the vascular function of the part—and disease of the heart or aorta will disturb the whole. But, it will be said, we have found a tumour in the brain, where there was paralysis of the feet—ergo, the former was the cause of the latter. We would answer, that tumours in the brain are every day seen without paralysis—and local paralysis is often found without any appreciable lesion of the brain or spinal marrow. Besides, how can a tumour in the brain cause paralysis in the legs and not in the thighs, seeing that the same nerves go to both parts?—The causes, then, of local or partial paralysis, we maintain, are often extremely obscure, and by no means to be always accounted for by cerebral or spinal diseases.

"Paraplegia dependent on the existence of disease in the brain, generally occurs at the middle or more advanced period of life than is usual in diseases of the bodies of the vertebræ, or their intervening fibro-cartilages. Its progress is more rapid than the slow insidious approach of symptoms from the latter diseases; the affection is more general, occasioning more or less paralysis of the upper and lower extremities, and this will often take place in a very few days from the occurrence of the complaint. This disease happens much more frequently in men than women. The gait of persons suffering from cerebral affection is peculiar, and very different from that attendant on affections of the spine. It very nearly resembles the vacillating steps of a drunkard.—Such paralytic persons are incapable of walking in a direct line; the limbs are loose, and thrown forward with an exertion of the whole body; there is a great consciousness of feebleness in walking, and the greatest difficulty in turning round. The appearance of the eyes often much resembles those of a drunkard, particularly when the patient is at all excited or anxious. The above analogy to the staggering steps of intoxication is readily understood, if we consider that it is the temporary disturbance of the brain, from the congestion of its blood-vessels, that deprives the drunkard of the power of directing his steps, and for the time induces a state bearing the closest resemblance to paraplegia.

"Sensation is more impaired than in spinal affections, when it will often remain perfect after a total loss of the locomotive powers. This impaired sensation is often peculiar, imparting an idea of some foreign body, as a leather glove or stocking, being interposed. The patient appears to feel, if I may use the expression, through a false medium; the limbs are more wasted and flabby, without any spasmodic rigidity of the muscles, which so often occurs in affections of the spine. Although often accompanied with a torpid state of the bowels, aggravated no doubt by the impaired muscular power of the abdominal parietes, there has not, in any instance that I have witnessed, been any train of gastric symptoms similar to those which so constantly attend affections of the spine, especially of the dorsal region. In some instances there is the additional confirmation of an impaired state of some of the external senses, accompanied with vertigo, a sense of weight on the head, and a general disturbance of the cerebral functions. As disease advances, the power of the brain in transmitting its influence to the extremities becomes more and more circumscribed.—Thus I have known a tubercular affection of the pia mater, in the first instance, cause a numbness and loss of feeling in the feet, which has gradually extended until all four extremities were completely paralysed, and the muscles concerned in respiration at length refusing their office, death ensued."

A very similar train of symptoms occurs in other diseases of the brain—especially in membranous inflammation and effusion, of the chronic kind. Whenever disease has proceeded the length that has been described, one or more of the mental faculties generally suffer; but in slighter cases it is difficult to form a correct diagnosis, and yet, as Mr. Earle observes, it is of importance to know whether the disease be seated in the head or the spine, that we may not unnecessarily subject the patient to the sufferings arising from the application of caustic issues and setons to the spine, when the disease is in the head. Our author thinks that attention to the following circumstances has materially assisted him in forming a correct diagnosis. He is, therefore, inclined to lay considerable stress upon it.

"It is well known that when a nerve is stimulated or injured in any part of its course, the painful sensation is referred by the percipient mind to the sentient extremity of such nerve; the familiar instance of the pain referred to the extremity of an amputated limb, may be adduced in proof of this. The exact reverse of this takes place when there is a partial paralysis arising from morbid affection of the cerebral organs. Here the centre of the sensorial functions being impaired, it appears to be incapable of transmitting its influence to the extreme parts of the body, and thus the feet and hands gradually lose their sensation or power of motion, or both; and in such cases if the nerves supplying the limbs be irritated, *they will convey the impression of such injury only part of the distance down the limb, about as far as the commencement of the paralytic affection.* I have repeatedly examined cases of paraplegia from affection of the spine, and in no one instance have met with the same phenomenon, which I have therefore been induced to consider as diagnostic of the paralytic affection being dependent on disease of the brain or its membranes; which opinion has in several instances been confirmed by examinations after death, in which both brain and spinal marrow have been carefully investigated."

Curvature of the spine, combined with structural diseases of the brain, tends to puzzle the practitioner, where there is also paraplegia, as it induces him to suspect the spinal marrow as the seat of the injury. Simple curvature of the spine, however, is a very different disease from the angular curve produced by disease of the bodies of the vertebræ. In the former case, the whole spine is curved in the form of a half-hoop, in consequence of the debility of the muscles of the back, which are no longer capable of sustaining the weight of the trunk and viscera, and maintaining the erect posture.

"The test by which I have been in the habit of trying these cases is at once simple and satisfactory. If a person with such a stooping or incurvated state of the spine be placed on a horizontal plane, the back will immediately and spontaneously be restored to its proper form without causing any pain or distressing symptoms, which would certainly be produced by any attempt at extension of a diseased spine. The state of the back in these cases is similar to what occurs during sleep or after death. It takes the direction influenced by the gravitation of the viscera and upper part of the body; for be it remembered that the strength of the spine and of most other joints depends on the power of the muscles: this is readily shown by dividing the tendons which pass over a joint, which will immediately become pliant and flexible. The same occurs in acute rheumatism, when the muscles are no longer under the control of the will, and the joints are consequently loose and powerless."

A great number of cases are adduced by Mr. Earle, but several of them, we think, are very unsatisfactory. Thus, in the first case, that of a gentleman, there were unequivocal symptoms of cerebral disease, as intense head-ach, &c. Paralytic affections extended from the feet and hands towards the trunk of the body. "On stimulating the median and ulnar nerves, he was not sensible of pain much below the elbow." How did Mr. Earle stimulate these nerves?

On dissection, there was found hydrocephalus to a considerable extent, and the whole of the pia mater was studded with tubercles. The cervical portion only of the spinal marrow was examined, and no disease was found there, except an increase of fluid.

In the second case, that of a young woman, there was loss of sense, but not of motion, in her hands. Some time before death she had an apoplectic fit, succeeded by hemiplegia. Three scrophulous tubercles and other derangements were found in the brain, and one in the cerebellum softened down. No disease could be detected in the spinal marrow; but the cancellous structure of the bodies of the vertebræ was filled with cheesy deposit, so that they could be easily cut with a knife.

In the third case, the patient had been thrown from his horse and hurt his back. Six weeks afterwards he became affected with some loss of muscular power about the mouth and pharynx, succeeded by numbness about his feet, and a sense of weakness in his legs. This last symptom extended upwards until the lower half of the body was paralysed. The same state took place in the upper extremities. When he came under our author's care, he was in a very deplorable condition. The bladder and rectum were paralysed, and he had hardly any power of deglutition. By directing remedial measures to the head, under the impression that there was inflammatory action in its membranes, the patient slowly and *partially* recovered. It is evident that this case is not satisfactory, in respect to the precise seat of the organic lesion.

The fourth case was that of a man, who came into Bartholomew's Hospital with paraplegia, including paralysis of the rectum and bladder. He walked like a drunken man. On examination, no disease of the spinal column could be detected. But, as there was something about his eyes which led Mr. Earle to suspect disease of the brain, he was treated accordingly, and, under this treatment, he improved. It afterwards came out that he had received a severe blow on the temple, several months previously, against which he took no precautions. Two months after this accident, he felt a stiffness in his feet and ankles, which gradually spread upwards, as high as his loins, accompanied by defect of sensation. He had suffered a good deal in his head after the blow. Repeated small bleedings from the head—an active seton—low diet—mild aperients, constituted the remedies; and, under this plan, he slowly improved. This case goes to the support of PROBABILITY in Mr. Earle's views, but no farther.

The fifth case, was that of a youth of 15, who came into Bartholomew's Hospital for paralytic affection of the lower extremities. Five years previously he had been operated on for cataract in both eyes, and, by the subsequent inflammation, one eye was destroyed. The other experienced a similar fate about a year afterwards. Severe head-aches attended and followed this last catastrophe, and his lower extremities became weak, with shooting pains, and some involuntary contractions of those members. The symptoms of cerebral disease were very evident when he came into hospital, and the proper remedies were directed to the part. He was so much relieved as to be able to leave the hospital.

From the above cases it will appear very probable—and, indeed, it cannot be reasonably doubted, that cerebral disease may produce paralysis of the upper or the lower extremities—but these cases are very far from *proving* the position, for the reasons alleged under each case. Neither do they militate against the doctrine we maintain, that local paralysis may depend on local affection of the nerves, independent of any disease in the brain or spinal marrow. Even when the original injury is sustained by one or other of the above parts, and local paralysis—say paralysis of the feet and legs follows,—it is highly probable that the nerves of those parts paralysed, have experienced some organic change not cognizable by our senses. If there be sensibility in the thigh and none in the leg, is it not evident that the immediate cause of this insensibility of the leg must be in the portion of nerves distributed below the knee?

Of the cases of paraplegia dependent evidently on disease of the spine, we shall notice only one.

This was a corpulent man who was thrown from his horse, and fell on the sacrum. Little or no inconvenience was experienced at the time; and it was not till after many months, that he felt a difficulty in throwing his leg over his horse. This was followed by disposition to trip, and inability to raise his feet and measure correct distances. These symptoms gradually increased, until the motive powers of the lower extremities were entirely lost. Various means, depletory and counter-excitant, had been used under the idea, that the disease was in the spinal cord, but the paralysis advanced upwards, and at length the superior extremities lost their muscular power. The rectum and bladder escaped paralysis. During all this time (nearly two years) his general health was good—his mental faculties clear. Sensation continued perfect to the last in all the parts deprived of motive power. He died at length, apparently from want of muscular power to breathe. His intellect was unimpaired within ten minutes of his dissolution. Various medical men were consulted during this patient's illness—and various opinions were entertained, as a matter of course. The more general opinion was, that this was one of those cases described by Dr. Baillie. Our author was led to impute the cause to affection of the spine—indeed we cannot see on what kind of evidence the other party grounded their diagnosis, for there was not a single symptom of cerebral affection from beginning to end of the complaint.

*Dissection.* The bodies of the last two lumbar vertebræ were very prominent, and bridges of newly formed bone connected several of the lower dorsal vertebræ. Much bloody effusion issued when the spinal canal was opened—"and great congestion of vessels was found *exterior to the membranes, between them and the bony canal.*"

Ulceration of the posterior surfaces of the bodies had commenced at several points in the lumbar and dorsal spine, with a thick yellowish deposit covering the ulcerated parts. There was more fluid than usual between the dura and pia mater. The latter was very vascular. The medulla itself was very hard, as was the cellular texture of the bodies of the vertebræ. In the head, a small quantity of fluid was found in the ventricles—and the arachnoid, at the basis of the brain, was slightly milky.

There cannot be a doubt that, in this case, the paralysis was owing to chronic inflammation of the spinal column, affecting both the osseous and the soft structures.

The cases where both head and spine were found involved in disease, we must pass over. Some of our mercurial brethren, in the noble art of reviewing, will doubtless think us extremely clumsy in occupying four or five pages of close type with an article of fifty octavo pages, the analysis of which they would have compressed into one or two pages of large type. We much doubt whether such analytical reviews can give satisfaction to either the author or the public. We shall certainly continue our usual practice of giving a complete analysis of all articles on which we enter, and where the matter is of a practical nature. Where criticism merely is wanted, we shall probably be as brief and to the point as our neighbours.

There are three distinct kinds of reviews—the analytical, the critical, and what might be termed the TOM-TIT-ICAL. This last species is of modern invention. Thus, in a recent number of a celebrated cotemporary, Dr. Abercrombie's whole work was *dispatched* in three or four columns, although a single division of it cost us many days labour, and has occupied fourteen or fifteen pages of our present Fasciculus!

### 2. *Rupture of the Uterus not fatal.* By Dr. SMITH.

The utility of registering cases where rupture of the uterus has not terminated in death cannot be disputed, as such cases fortify the mind in the prognosis which, we all know, is imperiously demanded from the medical practitioner, on such trying occasions. It is the duty of Journalists, however, to divest such registry of all unimportant details, as greatly abridging their diffusion through medical society at large.

*Case.* Mrs. H. aged 40 years, the mother of nine children, was taken in labour in the seventh month; the membranes broke; the pains were sharp; the presentation natural. In consequence of symptoms which justified venesection, this was directed, but could not be effected, in consequence of the fatness of the arm. Eight or ten hours after the rupture of the membranes, the patient complained of severe pain in her back, and through the abdomen, "as if a sword had been thrust through her," followed by a considerable discharge of blood from the vagina, faintness, and sickness. The labour-pains ceased—the countenance sank—the pulse rose to 130, extremely feeble—the abdomen became irregular in shape, and tender on pressure. On examination, no part of the fœtus could be felt. In a subsequent examination, Dr. Smith distinctly felt a rent, about two inches from the os uteri, across the posterior part of the uterus, nearly three inches in length, through which two fingers could be readily passed. The edges of the rupture were extremely thin. A consultation was proposed, but Dr. W. and the attendant surgeon, Mr. Whatman, were obliged to act. Mr. W. endeavoured to introduce his hand into the uterus, but found a resistance which he could not overcome. The attempt was made by Dr. W. and Mr. Charles, (who had just arrived) without success, in consequence of an adhesion of the os uteri to the vagina under the pubis, the effect of inflammation in a former labour. The most formidable symptoms now supervened, and it was determined to dilate the os uteri by a slight incision through the hardened part, so as to allow the hand to pass. This was performed dexterously by Mr. Whatman, and the hand was introduced into the uterus, the feet brought down, and the body of the child delivered. There was difficulty with the head, and, therefore, it was perforated through the lambdoidal suture, when the delivery was readily effected, the placenta soon following. Very little hæmorrhage succeeded. During these operations, the patient was often on the brink of death, but was supported by wine, brandy, and other stimulants. A large opiate was properly administered after the delivery was completed. The patient continued in a very alarming state for two hours, when she gradually recruited. Abdominal inflammation followed, with its usual phenomena, and was treated in a very masterly manner. This treatment we need not detail. The patient perfectly recovered, and lives a monument of the triumph of art over an otherwise inevitably fatal accident. We would ask Sir Anthony Carlisle what his female accoucheurs would have done in such a case—and what chance there would be of effectual chirurgical aid being given, if the practice of midwifery were confined to females? Why is the Westminster Hospital not left to the nurses, except when a surgical operation is to be performed? In midwifery, as in surgery generally, it is necessary to be practically acquainted with the efforts of Nature, in order to know when and how art is to interfere. If, therefore, you leave all natural and difficult labours to females, it will be nearly useless to call in surgeons or physicians when the case requires an operation of manual dexterity. But the question is absurd, and we are almost ashamed of alluding to it. It is the veriest chimera of a disturbed sensorium.

